# Postmenopausal Motherhood Reloaded: Advanced Age and In Vitro Derived Gametes

**DOI:** 10.1111/hypa.12151

**Published:** 2015-03-07

**Authors:** Daniela Cutas, Anna Smajdor

## Abstract

In this paper we look at the implications of an emerging technology for the case in favor of, or against, postmenopausal motherhood. Technologies such as in vitro derived gametes (sperm and eggs derived from nonreproductive cells) have the potential to influence the ways in which reproductive medicine is practiced, and are already bringing new dimensions to debates in this area. We explain what in vitro derived gametes are and how their development may impact on the case of postmenopausal motherhood. We briefly review some of the concerns that postmenopausal motherhood has raised—and the implications that the successful development, and use in reproduction, of artificial gametes might have for such concerns. The concerns addressed include arguments from nature, risks and efficacy, reduced energy of the mother, and maternal life expectancy. We also consider whether the use of in vitro derived gametes to facilitate postmenopausal motherhood would contribute to reinforcing a narrow, geneticized account of reproduction and a pro-reproductive culture that encourages women to produce genetically related offspring at all costs.

Motherhood after the age of menopause, facilitated by assisted reproductive technologies (ARTs), has raised much controversy in recent decades. To date, pregnancy in postmenopausal women has required the use of donated oocytes, for two main reasons. First, it is a complex task to freeze oocytes (due to their high proportion of water); until recently, most oocytes that were frozen were damaged in the process and failed to produce viable embryos (Van Blerkom and Davis 1994; Van der Elst 2003). With no means of storing eggs, once a woman ceases ovulating, she is dependent on donated oocytes if she wants to reproduce. Second, oocytes deteriorate with increasing maternal age, and the risk of genetic abnormalities in offspring when using one's own oocytes increases correspondingly (Lister et al. 2010). This risk can be obviated by using oocytes from a younger donor (even when the prospective mother still has her own).

Subsequent to recent developments, oocytes can now be preserved much more effectively than before, through vitrification (see, for example, Rienzi et al. 2012). However, oocyte preservation will be useful only if a woman knows of its existence, while she is still young enough to have healthy eggs, and has access to it. For those who are already heading toward age-related subfertility or menopause, the possibility of vitrification offers little hope.

Women in this situation, however, may be able to benefit from the capacity to generate eggs in vitro from other types of cells, either embryonic stem cells (ES cells) or induced pluripotent stem cells (iPS cells). Although in vitro derived gametes are not yet in use in humans, research is being carried out using nonhuman cells, with varying degrees of reported success. Research is ongoing with reprogramming of ES cells as well as iPS cells in labs across the world (see, for example, West et al. 2006; Heindrycks et al. 2007; Hinxton Group 2008; Atwood 2011; White et al. 2012). Both spermatogonial stem cells and oocytes have been generated from ES cells and iPS cells. Live mice were obtained both from oocytes and from sperm obtained ultimately from skin cells (Hayashi et al. 2011; 2012; Saitou in Cyranoski 2013)—and these went on to reproduce naturally, giving birth to healthy offspring.

It is difficult to predict whether, when, and how safely these technologies will be available for human use. Scientists themselves disagree on this (Cutas et al. 2014). Nevertheless, the realization of such a possibility could multiply the range of choices that prospective parents seeking to fulfill their reproductive wishes will have in the future. In this paper we will consider how the use in human reproduction of in vitro derived gametes may impact on the case for, or against, postmenopausal motherhood. We will assume here for the sake of argument that this will be technically feasible with a degree of safety comparable with that of other currently accepted reproductive technologies.^1^

If nonreproductive cells could be used successfully for the manufacture of in vitro derived eggs, menopause would no longer create the sort of infertility that it now does, and female fertility could be extended, in principle, indefinitely. Currently, the feasibility of postmenopausal motherhood is limited largely by its reliance on donated gametes. With the advent of in vitro derived eggs, this inbuilt limitation on the scope of postmenopausal motherhood would vanish. This leaves us with a number of ethical questions. Are we prepared for the increased scope in postmenopausal motherhood that might result from the development of in vitro derived eggs, or are there good ethical reasons to prevent women from accessing this technology beyond a certain age?

In 2012, a sixty-six-year-old Swiss woman gave birth to twins following IVF (in vitro fertilization) treatment undertaken in Ukraine. In 2009, Elisabeth Adeney gave birth to twins in England at the age of sixty-six. In 2006, Maria del Carmen Boussada de Lara gave birth to twins, in Spain, at the age of sixty-seven, following IVF treatment in the US where she lied about her age. In 2005, Adriana Iliescu gave birth to a daughter, in Romania, at the age of sixty-six. All of these women received treatment that was legal at the time in the countries where it was undertaken.

In response to these and similar cases, a number of arguments have been advanced relating to the use of ARTs for postmenopausal women. In the following, we will look more closely at some of these, and point out where in vitro derived gametes might have a significant impact. We will discuss the arguments from nature, risks and efficacy concerns, reduced energy of the mother due to age, maternal life expectancy, over-valuing genetic relationships, and the reinforcement of a pro-reproductive culture. (For more detailed discussions of arguments for and against postmenopausal motherhood, see, for example, Fisher and Sommerville /2003^1998^; Parks 1999; Pennings 2001; Goold 2005; Peterson 2005; Cutas 2007; Smajdor 2011.)

## Arguments from Nature

One of the perhaps most intuitive objections to postmenopausal motherhood is that it is “not natural.” Once women's biological clock has stopped ticking (because they have reached the age of menopause), we ought not to tamper with it. This, however, is a very tricky argument, vulnerable to objections in terms of the naturalistic fallacy: just because something happens naturally, it does not mean it should happen nor that we should not prevent it. Furthermore, the distinction between what is and is not natural is by no means straightforward.

In current debates on “postmenopausal motherhood,” the provision of treatment to women who are undergoing early menopause is not usually controversial. Rather, the problem seems to be the use of technology to allow women of advanced age to reproduce. A more precise formulation of what causes controversy is “motherhood after the *normal* age of menopause”: because what the objectors are concerned about is not the presence or absence of menstruation itself, but advanced age and questions of normality (Monbiot 2001).

Moreover, regulations are often also formulated in terms of age, rather than of menopause per se, thus making the concern with age explicit. In many legislatures, age limits are set for women seeking to use IVF (forty-five in Denmark, forty-two in France). Interestingly, these ages typically predate the average age of menopause by up to ten years (according to the UK's National Health Service, the average age of menopause is fifty-one) (NHS 2014). For the purposes of this paper, we will use the expressions “women after the age of menopause” and “postmenopausal women” or “postmenopausal mothers,” although we acknowledge that arguments for and against motherhood in these cases are also used in the case of premenopausal “older women”—and that objections to offering IVF to postmenopausal women are about their age rather than their lack of menstruation.

Another *unnatural* aspect of postmenopausal motherhood is the use of donor eggs. The problems with the use of donor eggs in these circumstances are that:

1. egg donation imposes burdens and risks on the egg donors—a problem that can be avoided once women can provide their own eggs; and
2. in this way the intended mothers give birth to children who are not their genetic offspring, raising objections from those who equate *real* motherhood with genetic motherhood—which, as we will see further on, may include the children and the prospective mothers themselves.

The ability to create eggs from the postmenopausal mother's own cells would clearly obviate the first objection, and would go some way toward mitigating the second. In a way, in vitro derived gametes would help postmenopausal women achieve a more *natural* motherhood: they would have *their own* genetic offspring. Nevertheless, even though a genetic link would be maintained, some might feel that postmenopausal motherhood still represents an unacceptable transgression of natural boundaries. Hub Zwart, for example, suggests that those who argue in favor of the postmenopausal woman's right to choose her own reproductive course fail to consider the “moral dignity of the body, of the biological nature of the person” (Zwart 1994, 73).

Of course, early menopause in younger women is also a natural biological phenomenon, in that it is not brought about deliberately by human intervention. Yet for Zwart there is a difference. He uses the analogy of a steel pin used to help bones fuse after a break. This, he argues, is not unnatural because it supplements the natural healing impulse of the body. The pin alone could not remedy the problem: the artificial and the natural work together. In postmenopausal motherhood, he suggests that this underlying natural impulse is absent. The postmenopausal woman's body (Zwart considers the case of a woman of fifty-nine) does not “want” to get pregnant, in the way that the pinned bone “wants” to heal. Postmenopausal motherhood is thus entirely “technical and artificial”; it instrumentalizes the body instead of working with it. In contrast, Zwart's argument implies that using the same technology to help a younger woman conceive fulfills this moral requirement for the artificial and natural to work together. However, natural conception, followed by successful gestation and birth, has been documented in a fifty-nine-year-old (Farmer 2007). This being the case, Zwart's argument that providing fertility treatment to fifty-nine-year-olds is inherently and intrinsically unnatural is unconvincing.

## The Need for Eggs

A variant of the argument from nature is the idea that whereas younger women “need” fertility treatment, we cannot construe postmenopausal women as having such a need because it is not natural for them to be able to reproduce. On this basis, treatment is acceptable for young women undergoing premature menopause, but not for women past the *normal* age of menopause.

Currently, many of the arguments related to postmenopausal motherhood conflate issues of medical need with the scarcity of donated eggs. Postmenopausal women seeking treatment are to some extent perceived as competing with younger women for access to the supply of donor eggs. Competition for access to oocytes has thus been an integral part of their use in ARTs. In this situation, it may seem appropriate to favor the claims of younger women, who may have undergone premature menopause, or may have suffered from fertility-affecting cancer, or may suffer from a genetic condition that they do not wish to pass on.

A postmenopausal woman, on the other hand, is simply attempting to override biology. It is normal for a woman of fifty-five not to have viable eggs. Therefore, her claim to *need* oocytes may be regarded as less compelling. In addition to this, there may be a perception that the postmenopausal mother does not *deserve* access to this scarce resource, since she could, or should, have chosen to reproduce while younger. She has chosen to delay childbearing, and is thus responsible for her situation in ways in which the younger claimant is not (Bewley, Davies, and Braude 2005).

These assumptions about need and desert in older and younger patients are of course open to question (Smajdor 2008). However, the important point here is that the arguments they generate are entirely dependent on the additional fact that oocytes are scarce. If the oocytes can be procured from the patient herself, we will be looking at a different set of arguments and concerns, in which the scarcity of donated oocytes, and the burdens of oocyte donation, no longer play a part.

## Risks and Efficacy

Postmenopausal motherhood involves higher risks of miscarriage, premature birth, C-section, and for the child, the loss of their mother early in life. These risks have been discussed in detail by the authors cited above. Currently, postmenopausal motherhood is open only to women who are either willing to use, and able to access, donated eggs, or who have preemptively preserved their own eggs. However, the use of in vitro derived gametes could significantly increase the number of women who might consider postmenopausal motherhood. Even if the risks of postmenopausal motherhood to the woman herself are no greater, when in vitro derived gametes are used, than those associated with other means of postmenopausal motherhood, the larger numbers of women being exposed to these risks might give cause for concern.

From the perspective of the woman herself, the avoidance of risk is not always or straightforwardly the best course of action. Indeed, if it were, we would advise women of *any* age to avoid pregnancy and childbirth, since these are risky endeavors whatever a woman's age, compared to not being pregnant and not giving birth (Grimes 1994). Another important consideration here is the question of autonomy and paternalism. It is a central tenet of medical ethics that adults can make decisions that may be regarded by others as foolish or reckless. Only where there is serious danger to people other than the risk-taker is intervention legitimate.

Clearly, in the question of older motherhood, there *are* others who are affected: the offspring most directly, as well as the rest of society. One of the most obvious causes of potential harm both to offspring and to society in general is the death of the mother while her child is still an infant. Aside from the emotional and psychological distress caused to the child, there may be significant social costs in terms of supporting it. So it is crucial here to consider whether these risks are of a degree significant enough to warrant state prohibition. If the answer to this question is “yes,” then we also need to consider what kinds of intervention are justifiable.

One effect of the availability of in vitro derived gametes for reproduction after the age of menopause might be that the new possibilities they bring about would incentivize even more women to postpone motherhood. This in turn would subject them to higher risks during pregnancy and childbirth, and would impose costs on society. Although this may be the case, it is not clear that this consideration justifies restricting the use of new ARTs. It is possible that this option will benefit women in the long run, and that it will allow more women to eventually become mothers who would not have done so when younger anyway. The possibility to delay motherhood in this way may also allow children to be born to more confident mothers, who have not had to make this step any earlier than they felt they were prepared for it (Goold and Savulescu 2009; Mertes and Pennings 2012).

## Welfare of Offspring

### Maternal Energy

Bringing up a child requires energy. It might seem reasonable to claim that when people seek treatment, their capacity in this respect should be taken into account. However, should age alone be the delimiting criterion? It is not obvious that a(ny) sixty-year-old is incapable of bringing up a child. In the days of high maternal mortality, it was very common to be brought up by one's grandparents. Today also, grandparents can play a significant part in childcare and child-rearing. Furthermore, in the words of Fleur Fisher and Ann Sommerville,

[m]any women over 60 are caring, with little support, for even older parents with multiple problems, such as incontinence and dementia. Such demands take a toll on carers which is arguably as great or greater than caring for a child. (Fisher and Sommerville /2003^1998^, 213)

However, it might still be argued that older people are likely to have less stamina, be less robust, and are more exposed to health risks, which considerably impair the continuous effort that is required in bringing up children. And although grandparents may shoulder some or all of the responsibilities of parenthood, perhaps this is a suboptimal and often temporary arrangement that we should not bring about deliberately. Older parents may be less resilient, less active. They may be unable to engage in the games and rough-and-tumble that children love and from which they benefit in many ways.

There are two important considerations to be addressed when attempting to determine how far physical frailty should count against prospective postmenopausal mothers. As with the specific problem about age and life expectancy, frailty is not the sole domain of the elderly. Disease and disability affect the physical capacities of many parents, and may be among the reasons for seeking fertility treatment (Parks 2009). A young, wheelchair-bound mother will not be able to run around with her children. A sufferer of early onset arthritis may be in too much pain to engage in much boisterous physical interaction with her children. But would we conclude that these mothers should therefore not have (had) children? If not, it seems that unfair discrimination is at play. If it is unacceptable to be put in a situation in which one's mother walks slowly with the aid of a stick, this must surely be so whether the mother is thirty-five or fifty-five.

There is a common tendency to exceptionalize motherhood: to regard the mother of a child as necessarily the only person who can or should perform all the tasks and activities involved in childcare, and the only person who can or should meet all the child's needs. Children, however, do not live in a vacuum with their mother: they are part of a social world in which many people may participate and contribute to the growing child's experience (Mullin 2005). It is nice if mothers can run around with their children, but if they cannot do this, there may (and perhaps should) be someone else in the child's life who can.

### Maternal Life Expectancy

Life expectancy alone does not say much about any particular person. If postmenopausal women interested in using IVF pass the health check that fertility centers undertake, then already they are likely to be healthier than the statistical average. The problem here might be with reckless fertility centers that would do anything for money. Over the long term, such a strategy would be detrimental, as their success rates would suffer significantly. Ultimately, this is a practical risk that can be managed with regulation—regardless of the age of the clients. After all, this is not specific to older patients but to patients with suboptimal health in general.

With access to good medical care, it is likely that someone who becomes a parent at sixty will live to see her child or children become adults (Mori 1995; Fisher and Sommerville /2003^1998^). Should we demand more for children than a statistical likelihood of their mother surviving until they are in their twenties? David Banh, Dara Haveman, and John Phelps suggest that a general rule of thumb should be that patients have a life expectancy of at least eighteen years (Bahn, Haveman, and Phelps 2010). This seems reasonable, in that in other respects our society deems eighteen-year-olds to be independent adults. Children who lose their parents during childhood will require alternative parenting arrangements; the burden of their care will arguably fall upon others, and it can make sense to avoid the facilitation of such situations.

As adults, we can expect to lose our parents at some point; later is preferable to sooner, and indeed, some people struggle to recover from the death of a parent, even in middle age (Marks, Heyiung, and Song 2007). But given that we all face losing our mothers during adulthood, it is not obvious that losing one's mother at twenty-two is a personal or social tragedy of such magnitude as to query the morality of having been brought into existence—any more than losing one's mother at thirty-two, forty-two, or later.

If it is acceptable to enforce life expectancy requirements for prospective parents, it is not clear why it should be enforced only in cases of prospective menopausal mothers. Many of those seeking IVF or other fertility treatments may face limited life expectancy, so if we accept Banh, Haveman, and Phelps's points, we should apply the same criteria to *all* prospective parents, regardless of age. In some cases, having undergone treatment for cancer or other conditions may be the key reason for seeking fertility treatment. It may then seem equally appropriate to inquire into the life expectancy of such patients. After all, there seems to be no reason why it is preferable to lose one's thirty-five-year-old mother to cancer than to lose one's eighty-five-year-old mother to old age.

Although it may seem desirable that people should have children only in optimal circumstances, in practice it is very difficult to determine what optimal circumstances really are, and to draw up limits that are both just and practicable. Many of the arguments formulated against older mothers appear to be based on concern for the offspring, but on closer inspection they reveal distaste or prejudice against older mothers. If, for example, having a likely lifespan of only fifteen years makes it unacceptable to have assistance in becoming a parent, then this is also unacceptable in the case of people who might have a medical condition that reduces their lifespan, and perhaps also in the case of firefighters, people in the army, or people who take part in extreme sports that may put them at greater risk of premature death—at least as long as they do not change these circumstances (Cutas 2007).

Ethnicity, geographical location, occupation, education, and socioeconomic status also affect life expectancy. A black woman in the US has a lower life expectancy than a white woman. Yet lower age limits are not imposed for black women seeking fertility treatment. Similar points can be made about poverty and educational attainment. Research suggests that the life expectancy gap between the most and the least educated women is more than ten years (Olshansky et al. 2012). The idea that fertility treatment could be withheld from women on these grounds raises concerns—and rightly so. The reason for this is straightforward: we should not discriminate on the basis of ethnicity, wealth, or educational attainment. However, discriminating on the basis of age alone seems more acceptable. The putative distinction between acceptable and unacceptable forms of discrimination in this context is highly problematic.

## Overvaluing Genetic Parenthood

Because of the promise to maintain the genetic link between mother and child, in vitro derived gametes might make motherhood more appealing for some women who might not have thought about parenting anymore, since they would not be able to have *their own* genetic children. At the same time, the possibility for postmenopausal women to become mothers in the genetic sense will neutralize some of the objections to postmenopausal motherhood: that by using ARTs, these women do not become *real* mothers, but they are the birth (if they carry the pregnancy) and social mothers of other women's genetic children; and that older women's reproductive projects require other women to subject themselves to the discomforts and risks of oocyte donation.

Though not often explicit in objections to postmenopausal motherhood, the undercurrent of the mistrust of such reproductive projects because of the lack of the genetic link between mother and child has been very pervasive in media reports. This is a sensitive area of heterologous reproductive technologies, which is salient not only for objectors but also for the parents, who have to work toward representing themselves as their children's “real” parents, and for their children who have to understand the dissonance between their genetic parents, their birth parents, and their social and legal parents (Mac Dougall et al. 2007; Gurnham 2012).

A possible side effect of the use in reproduction of in vitro derived gametes is that it may help to reinforce attachment to genetic parenting at any cost. This may be mitigated by awareness-raising and in general by more discussion of the complex aspects of parenting. Being given yet another choice to become a genetic parent does not necessarily lead to more genetic essentialism or attachment to genetic relatedness. Moreover, in conditions of scarcity of available oocytes, in vitro derived gametes may lessen the burden on women to donate oocytes, and may simply create more opportunities for women to become parents, even if, incidentally, of their own genetic children.

A chance to maintain a genetic link between parent(s) and children is one of the reasons why people choose ARTs in the first place—but not the only one. Other reasons may be that, at least in some cases, ARTs allow the prospective mother to carry a pregnancy even if she cannot conceive naturally, or that ARTs are easier to access than adoption services. Indeed, in the case of postmenopausal women, in several European states they may be able to use ARTs while being beyond the eligible age for adoption services. In the UK, for example, there is no statutory upper age limit for IVF nor for adoption. However, individual adoption agencies can impose their own age limits, which are not always openly publicized. According to UKadoption.com, the cut-off is usually between thirty-five and forty (UKadoption.com).^2^ Effectively, therefore, a woman may be able to access fertility treatment after her eligibility for adoption has passed: a regulatory inconsistency that, in those legislatures, drives people toward using ARTs when they could adopt instead with lower costs to everyone involved. This is further aggravated by the valuing of the creation of children as a necessary component of becoming a parent and the subsequent perception of adoption as a viable choice only for when all else has failed (Bartholet /1999^1994^; Brakman and Scholz 2006).

It may become harder to argue against access to treatment if that treatment can produce genetically related offspring, as opposed to unrelated offspring. Once the means become available, a strong case can be made. The arguments about clinical need are difficult to articulate or defend, but other arguments based on justice and equality or reproductive rights may be harder to defeat. If men can have genetically related children at any age, it becomes a matter of justice that women should be able to do so as well. If some women can do so, it becomes a matter of justice for *all* women. If there is a right to reproduce, and that right has some kind of genetic or biological component, it is easier to construe access to in vitro derived gametes as meeting that right as opposed to, for example, donated eggs or surrogacy.

The use in reproduction of in vitro derived gametes has the potential to allow more reproductive equality between women and men. Whereas men usually retain their fertility until later ages, and have had for decades the choice to store their sperm for future use, women lose their fertility at menopause and until recently have had far more meager prospects for storing their oocytes. Thus, although this inequality was not unfair so long as there was nothing we could do about it, fairness may require that women be allowed the same choices as men as soon as these choices have become feasible.

In most Western societies and beyond, the genetic link between parents and children is considered a valuable component of parenting. In the context of changing regulations around gamete donation, parents of resulting children may have to consider difficult conversations with them about their genetic origins, and handle all related issues. Having one's own offspring can reduce the number of people involved. And, more generally speaking, having their own genetic children would allow postmenopausal women to recognize their own family traits in their children, as can all people who reproduce naturally.

Although the presence of the genetic link is sometimes seen as the ultimate marker of parent–child connections, as a necessary, or at least as a desirable component of such relationships, there are also those who warn about the negative effects of the unquestioned attachment to it. It has been argued that the belief in the power of this type of connection encourages some parents to see themselves as somehow the “owners” or the “sovereigns” of their genetic offspring (LaFollette 1980; Archard 2004, 9–10, 142–45; Brakman and Scholz 2006, 60). According to some authors, the emphasis on the genetic link to one's children is not only misguided but also harmful, and betrays “an unwarranted emphasis on the importance of biological as opposed to social connectedness” (Levy and Lotz 2005, 243). If the reason why people want to have genetically related children is that these would represent the fulfillment of their mutual “love, trust and commitment,” then they are mistaken, because it is not the genes of their children that fulfill that love. Rather, “it is the decision to parent together that expresses the mutual love, trust, and commitment of partners in a relationship” (246)—and, indeed, parenting itself need not include any particular degree of genetic relatedness. Moreover, their argument goes, the genetic claim reinforces proprietorial conceptions of parent–child relationships: my genetic child is *more mine* than any other child that I may raise. The argument by Neil Levy and Mianna Lotz thus translates an advantage of some ARTs—providing people with the possibility to have genetic offspring—to a disadvantage and an objection to the encouragement of genetic essentialism that it brings.

A growing body of research indicates that the presence of the genetic link between parents and children presents no guarantees, and that at the same time its absence does not impair the functioning of parent–child relationships (Golombok et al. 1995; Chan, Raboy, and Patterson 1998; Golombok 1998; 2000; Scheib and Hastings 2012)—and even that parents do better at parenting in the absence of genetic relatedness with their children (Hamilton, Cheng, and Powell 2007).

Notwithstanding the debate around the goods and bads of the genetic link, the implementation (if possible) of the desire to integrate the genetic factor *too* into the parental project cannot be fairly dismissed unless we are prepared to address the status quo and correct the negative implications of there being so much value placed on it. And lastly, with or without the genetic link, in vitro derived gametes may be the only chance that a woman has to become a mother (for example, because she is not eligible or there are no available children to adopt, scarcity of donated oocytes, and so on)—in which case not allowing her access effectively prevents her from becoming a mother at all.

## Encouraging a Pro-Reproductive Culture, and Never Giving Up

Throughout their lives, women are subjected to conflicting messages about the appropriateness of their reproductive choices, and are not encouraged to be sensitive to reasons and question beliefs and expectations about reproduction (Bortolotti and Cutas 2009). While becoming a parent *naturally* is largely unregulated, access to assistance (either social or medical) often is, and sometimes very strictly so. For example, in some countries only married and/or heterosexual couples are eligible. In others, gamete donation is forbidden. In some countries, there are age restrictions. In most countries, a child's parents are by default her birth mother and her husband if she has one—a criterion that leaves out same-sex parents and creates a host of problems for ARTs users and their collaborators. ARTs not only multiply and diversify types of contributions to creating children, but have for the first time allowed the splitting of biological motherhood into two (the genetic and the birth mother), and perhaps three (in the case of mitochondrial transfer—if we see the contribution of the mitochondrial DNA donor as amounting to genetic motherhood). Innovations such as these, fueled by people's quest to become parents, challenge current regulatory regimes as well as policy-makers and the general public: what does it mean to reproduce? Which of the individuals who contributed to a child's creation are her parents?

The availability of contraception has allowed people some control over the timing and number of their children, converting parenting into a choice. At the same time, however, womanhood and motherhood “continue to be conflated and mutually defined,” and women's failure to reproduce is often perceived as a failure to achieve their natural potential (Lewin 1993, 191). Voluntary childlessness has been and still is seen as a sign of immaturity or selfishness, and childless women are often seen as “psychologically maladjusted, emotionally immature, immoral, selfish, lonely, unhappy, unfulfilled, sexually inadequate, unhappily married, and prone to divorce” (Veevers 1980, 7), “deviant,” “aberrant, immature, and unfeminine” (Gillespie 2000, 225; Letherby 2002, 10). Such negative perceptions of childless women may have subsided to some extent in some environments but are still shared today (Vinson, Mollen, and Smith 2010; Peterson and Engwall 2013). Failure to reproduce is questioned, and “[p]eople keep asking childless women why they are childless, whether they plan to remain childless, how they feel about being childless, and they warn them that they are going to be lonely in their old age” (Stotland in Rhodes 2003).

In such a context, it is not surprising that women may feel encouraged to do whatever it takes to become parents. Indeed, talking specifically about the prospect of in vitro derived gametes, representatives of ART patients have expressed worries that the introduction of new technologies impels women to keep on trying: “[s]ociety gives you the pressure that if you want to have a child you have to do everything” and “[i]f you don't do everything, you don't want hard enough. The more possibilities there are, the more pressure on people to try everything” (Cutas et al. 2014). Concerns such as these are not new and fit into a broader set of criticism of ARTs by feminist authors (see, for example, Spallone and Steinberg 1987; Brakman and Scholz 2006, 63) for their potential to further stunt rather than expand women's autonomy.

At the same time, however, motherhood after the age of menopause is unpopular, and in some countries, such as France (Legifrance 2011, Art. 33) and Italy (Parlamento Italiano 2004, Art. 5), it is illegal to assist women after a certain age to access ARTs. Women, therefore, have to do anything it takes to become mothers, but not after a certain age. The combination of such conflicting messages, both from society at large (*try everything, you are not a whole woman if you don't, but once you have reached a certain age, don't try*) and from innovators (*here you have yet another way*), can hardly be presented as empowering women. However, although concerns such as these are important and must be raised in any discussion of women's reproductive autonomy, they are not intrinsic to any particular innovation. There is, moreover, no contradiction between developing and introducing new forms of ARTs and the autonomy of women's choices, between allowing reproductive choice while at the same time questioning pro-reproductive assumptions. Saying that new technologies should not be introduced because they encourage women to try too much skips the important step where all the other societal messages come in. Denying women a possibly viable and perhaps the only method available to them for becoming mothers, while they are subjected to a pro-reproductive culture that accords them attributes such as those mentioned above for not being mothers, may not be the most efficient or fair tool against said culture.

## Which Prospect?

A number of responses are possible to questions about the ethics of using in vitro derived gametes to enable women after the age of menopause to become mothers. Policy-wise, if there already are regulations in place preventing women after a certain age from accessing ARTs, then these women will not have access to IVF with in vitro derived gametes. Depending on why women over a certain age are denied access, the introduction of in vitro derived gametes as one type of ART might change this status quo: for example, if the policy is based on concerns related to scarce resources (oocytes) or exploitation of egg donors.

Societal expectations of men and women and of mothers and fathers are changing. News reports of postmenopausal women becoming mothers are now less sensational. These changes and this normalization are bound to have an impact on how the choice of postmenopausal motherhood is received in our societies, just as our habituation to ARTs has diminished the moral anguish associated with their use.

The prospect of the successful development of in vitro derived gametes for reproductive purposes brings about a series of changes that require further reflection and regulatory revisions. Whether this is good news in the realm of postmenopausal motherhood will depend to a great extent on the underlying reasons for opposition to allowing it. In vitro derived gametes might at the same time strengthen the case and the hope of prospective postmenopausal mothers, thereby making it even more difficult to resist demand and to justify the resistance in a fair way.

This takes us to the question of scope: we do not know what percentage of women who seek ARTs are beyond the age of menopause, nor can we predict how many might be incentivized to seek treatment. Arguably, the existence of non-age-based access restrictions, such as a minimum threshold of health status for responsibly administering fertility treatments, would prevent most prospective postmenopausal mothers from accessing the technologies anyway—even assuming a surge of interest.

Nevertheless, one opportunity that the prospect of in vitro derived gametes offers—regardless of whether they ever become a viable fertility treatment—is to provide us with a tool to review the reasons and justifications for our ethical evaluations and for policies regarding the use of ARTs. By presenting us with the prospect of giving postmenopausal women the unprecedented possibility to reproduce genetically, in vitro derived gametes highlight the progress of ARTs and their increasing capacity to allow more multifaceted parenting (not only legal and social and gestational, but genetic also) and the need to examine the forces behind the pursuit of genetic parenting at all costs—and to take steps to reduce the effects of undue pressure on prospective parents.

## Notes

1 Questions may be raised about risks to offspring from such experimental procedures. We cannot address these within the scope of this paper. Our focus here is an analysis of the issues that would arise within whatever safety-based parameters had been set.

2 The link was no longer accessible by the time we submitted this article. Policies and practices around adoption change, as well as those around fertility treatments: but this does not affect our claim that these policies and practices are formulated and enforced largely independently of each other, and that access to fertility treatments may be easier to obtain by some prospective parents than access to adoption.
